# Differences in neuroinflammation in people who started antiretroviral treatment during primary versus chronic HIV infection: an 18kDa Translocator protein (TSPO) positron emission tomography (PET) study

**DOI:** 10.1007/s13365-024-01200-3

**Published:** 2024-04-04

**Authors:** Jasmini Alagaratnam, John P. Thornhill, Zhen Fan, Jaime H. Vera, Jonathan Underwood, Rebecca Hall, Graham Searle, David Owen, Paul Edison, Sarah Fidler, Alan Winston

**Affiliations:** 1 Department of Sexual Health & HIV, Chelsea & Westminster Hospital NHS Foundation Trust, London, UK; 2 Department of Infectious Disease, Faculty of Medicine, Imperial College London, London, UK; 3 Blizard Institute, Barts & the London School of Medicine & Dentistry, Queen Mary University of London, London, UK; 4 Invicro, A Konica Minolta Company, London, UK; 5 Department of Global Health and Infection, Brighton and Sussex Medical School, London, UK; 6 Division of Infection and Immunity, School of Medicine, Cardiff University, UHW Main Building, Heath Park, Cardiff, CF14 4XN UK; 7 Department of Brain Sciences, Imperial College London, London, UK; 8 Department of Genitourinary Medicine & HIV, St Mary’s Hospital, Imperial College Healthcare NHS Trust, London, UK

**Keywords:** HIV, Neuroinflammation, TSPO, PBR28, Primary HIV

## Abstract

Persistent inflammation is described in people with HIV (PWH) on antiretroviral treatment (ART). Early ART initiation is associated with reduced inflammation. We aimed to evaluate neuroinflammation, using translocator protein (TSPO) [^11^C]PBR28 PET neuroimaging in PWH who initiated ART during acute HIV (aPWH) versus chronic HIV infection (cPWH) versus a control population. This was a cross-sectional, observational study. All participants underwent [^11^C]PBR28 PET-CT neuroimaging. Using a two-tissue compartment model, total volume of distribution (V_T_) and distribution volume ratios (DVR) using cortical grey matter as a pseudo-reference region at 20 regions of interest (ROIs) were calculated. Differences in V_T_ and DVR were compared between groups using the Kruskall-Wallis test. Seventeen neuro-asymptomatic male PWH on ART (9 aPWH, 8 cPWH) and 8 male control participants (CPs) were included. Median (interquartile range, IQR) age was 40 (30, 46), 44 (41, 47) and 21 (20, 25) years in aPWH, cPWH and CPs, respectively. Median (IQR) CD4 (cells/µL) and CD4:CD8 were 687 (652, 1014) and 1.37 (1.24, 1.42), and 700 (500, 720) and 0.67 (0.64, 0.82) in aPWH and cPWH, respectively. Overall, no significant difference in V_T_ and DVR were observed between the three groups at any ROIs. cPWH demonstrated a trend towards higher mean V_T_ compared with aPWH and CPs at most ROIs. No significant differences in neuroinflammation, using [^11^C]PBR28 binding as a proxy, were identified between cPWH, aPWH and CPs. A trend towards lower absolute [^11^C]PBR28 binding was seen amongst aPWH and CPs, suggesting early ART may mitigate neuroinflammation.

## Introduction

With the widespread implementation of modern antiretroviral treatment (ART), the prognosis and life expectancy for people with HIV has significantly improved (Hogg et al. 1998; May et al. 2014). However, people with HIV on virologically suppressive ART remain at increased risk of non-AIDS co-morbidities including cognitive impairment (Robertson et al. 2007; Schouten et al. 2014; Simioni et al. 2010). The underlying mechanisms are likely multifactorial; persistent microglial activation (Anthony et al. 2005; Minagar et al. 2002) and neuroinflammation in people with HIV on virological suppressive ART (Edén et al. 2016) is postulated to be a contributor.

The 18kDa Translocator protein (TSPO), located on the outer mitochondrial membrane, is highly expressed in activated microglia (Banati et al. 2004; Liu et al. 2014) and is associated with neuroinflammation (Liu et al. 2014). Synthetic radiolabelled ligands that selectively bind to TSPO have been developed and can be utilised to image dynamic microglial activation in the human brain in vivo, using positron emission tomography (PET) imaging (Stephenson et al. 1995). The first-generation TSPO radiotracer, [^11^C]-PK11195 was limited by low blood–brain barrier penetration and high nonspecific binding. Second-generation radiotracers such as [^11^C]PBR28 and [^11^C]DPA-713 have improved blood–brain barrier penetration and signal-to-noise ratios, however binding affinity is dependent on single-nucleotide polymorphism rs6971, whereby genotypic testing must be carried out to ascertain whether an individual is a low-, medium- or high-affinity binder.

TSPO PET brain imaging has been used to investigate neuroinflammation in people with HIV (Boerwinkle et al. 2020; Coughlin et al. 2014; Garvey et al. 2014; Hammoud et al. 2005; Vera et al. 2016; Wiley et al. 2006), with conflicting results. While higher TSPO binding in people with HIV has generally been reported compared with persons without HIV, the precise anatomical locations have varied between studies. These inconsistencies are likely related to (1) different methodologies used to quantify TSPO radiotracer uptake, (2) cohort differences (inclusion or exclusion of people with HIV with cognitive impairment), (3) differences in criteria for defining cognitive impairment (Nightingale et al. 2021) and (4) small sample sizes.

Strategies to reduce persistent immune activation and inflammation include the early initiation of ART, soon after HIV acquisition (Pace & Frater 2014). Early ART initiation may partly mitigate the effects uncontrolled HIV replication has on the central nervous system and thereby reduce the persistent neuroinflammation which has been observed in ART-treated individuals. Early evidence supports a reduction in cerebrospinal fluid biomarkers of inflammation when ART is initiated during acute HIV infection (Hellmuth et al. 2019; Oliveira et al. 2017), however, long-term data on brain parenchymal inflammation and neurological sequelae are lacking.

The aim of this study was to evaluate neuroinflammation, measured using TSPO [^11^C]PBR28 radiotracer binding, in people with HIV who initiated ART during acute versus chronic HIV infection, and compared with control individuals, using volume of distribution (V_T_) as a primary outcome and distribution volume ratio (DVR) as a secondary outcome.

## Methods

All participants underwent structural cerebral magnetic resonance imaging (MRI) and cerebral positron emission tomography-computed tomography (PET-CT) imaging with [^11^C]PBR28 ligand at the Imanova Centre for Imaging Sciences, London, UK.

### Participants living with HIV

Individuals attending HIV outpatient services at Imperial College Healthcare NHS Trust, London, UK, were invited to enrol into the study. Eligible participants were adult males ≥ 18 years of age, receiving ART for ≥ 3 months with plasma HIV RNA < 50 copies/mL, high affinity binders to [^11^C] PBR28 on TSPO genotypic testing and in good health. Exclusion criteria included significant neurological comorbidities, alcohol or recreational drug use disorder, contraindication to arterial cannulation, lumbar puncture or magnetic resonance (MR) imaging, body mass index > 30 kg/m^2^ and participation in another research study involving ionising radiation such that the subject would be exposed to a cumulative dose of > 10 mSv in the previous 12 months. Participants with HIV were assigned into two cohorts: participants who initiated ART during acute HIV infection were defined as people who had started ART within three months of confirmed primary HIV infection, based on one of the following six criteria: a) positive HIV-1 serology within a maximum of 24 weeks of a documented negative HIV-1 serology test result (can include point of care test (POCT) using blood for both tests), b) a positive p24 antigen result and a negative HIV antibody test, c) negative antibody test with either detectable HIV RNA or proviral DNA, d) Recent Infection Testing Algorithm (RITA) test reported as “Incident” confirming the HIV-1 antibody avidity is consistent with recent infection (within the preceding 16 weeks), e) weakly reactive or equivocal 4th generation HIV antibody-antigen test and f) equivocal or reactive antibody test with < 4 bands on western blot. Participants who initiated ART during chronic HIV infection were defined as people who had started ART more than six months after known or presumed date of HIV acquisition.

The studies were approved by UK Research Ethics Committees (REC) (reference numbers 16/LO/0096 and 12/LO/1570). Permission was obtained from the UK Administration of Radioactive Substances Advisory Committee (ARSAC) (reference numbers: RPC 630/3764/34269 and 630/3764/ 29,163) for the administration of [^11^C] PBR28. All participants provided written informed consent prior to commencing any study procedures.

### Control participants

Brain [^11^C] PBR28 PET-CT imaging data for the control participants were obtained from participants enrolled into either a human pharmacological blocking study to determine brain [^11^C] PBR28 binding potential in vivo(Owen et al. 2014) or enrolled into a brain [^11^C] PBR28 PET-CT imaging database for healthy volunteers supported by GlaxoSmithKline plc. The eligibility criteria to enrol into the pharmacological blocking study and the GlaxoSmithKline plc healthy volunteers’ database were similar to the criteria for the studies for persons with HIV.

### Brain magnetic resonance imaging (MRI)

T1-weighted whole brain structural images were obtained on a Siemens MAGNETOM® Verio 3.0 Tesla magnetic resonance scanner (Siemens Healthineers, Munich, Germany).

### Brain [^11^C] PBR28 positron emission tomography-computed tomography (PET-CT) imaging

1. Radiotracer synthesis
  [^11^C] PBR28 was produced on site at the Imanova Centre for Imaging Sciences immediately before use, according to local standard operating procedures. Quality assurance assessments were made using validated procedures in accordance with good manufacturing practices before injection to ensure the manufactured [^11^C] PBR28 met the prerequisite specifications
2. Arterial blood sampling and processing
  Following skin infiltration with 1% lidocaine as local anaesthetic, a cannula was inserted into the participant’s radial artery to enable regular arterial blood sampling throughout the cerebral PET-CT imaging procedure.
3. PET imaging data acquisition
  The cerebral [^11^C] PBR28 PET-CT images were acquired using a Biograph 6 PET-CT scanner (Siemens Healthcare). Participants were injected with an intravenous bolus of [^11^C] PBR28 over 20 s at the beginning of the 90-min 3D-mode of dynamic PET acquisition; injected activities ranged from 120.45 to 374.45 MBq.
4. Blood data processing
  Arterial blood was sampled to enable generation of an arterial plasma input function. A continuous sampling system (ABSS Allogg, Mariefred, Sweden) was used to measure whole blood activity each second for the first 15 min of each scan. Discrete arterial blood samples were manually withdrawn at 5, 10, 15, 20, 30, 50, 70 and 90 min time points after the scan commenced to measure whole blood and plasma activity. Samples obtained at 5, 10, 20, 30, 50, 70 and 90 min time points after the scan commenced were analysed using high-performance liquid chromatography (HPLC) to determine the fraction of parent radioactivity in the arterial plasma. The first three discrete blood samples were used to calibrate the continuous blood data, then the continuous and discrete datasets were used to form a whole blood activity curve, covering the duration of the scan. Discrete plasma samples were divided by the corresponding whole blood samples to obtain the plasma-over-blood (POB) data.

An exponential approach to a constant POB model was fitted (Gunn et al. 2000), to generate the metabolite-corrected plasma input function. This POB value was then multiplied by the whole blood curve to generate a total plasma curve. A sigmoid model was used to fit the parent fraction data.

The resulting fitted parent fraction profile was multiplied by the total plasma curve and then smoothed post-peak using a tri-exponential fit to derive the required parent plasma input function. For each scan, a time delay correction was fitted and applied to the input function to account for any time delay between blood sample measurement and the tomographic measurements of the tissue data. Free fraction in plasma (f_p_) was measured through ultrafiltration (Amicon Ultra regenerated cellulose MWCO 30 kDa, Millex, Ireland) in triplicate using Tris buffer (0.1M, pH = 7.4) to determine and enable correction for non-specific binding.

### [^11^C] PBR28 positron emission tomography-computed tomography (PET-CT) data processing

All brain [^11^C] PBR28 PET-CT data from the participants were analysed using the MIAKAT™ v4.3.17 pipeline via the same process. PET data were reconstructed using filtered back projection, corrected for attenuation and scatter (based on low-dose CT acquisition scan). Dynamic data were binned into 26 frames (durations: 8 × 15 s, 3 × 1 min, 5 × 2 min, 5 × 5 min and 5 × 10 min). Motion correction in the dynamic PET data was performed via frame-to-frame image registration of the non-attenuation corrected PET image to the participants’ structural T1-magnetic resonance image using SPM5 (Wellcome Trust Centre for Neuroimaging), with a mutual information cost function.

The CIC neuroanatomical atlas version 2.0 was nonlinearly deformed into the participant’s space, via structural T1-MRI data mapping, to obtain a personalised anatomical parcellation of regions of interest (ROIs). The following ROIs were chosen to assess levels of binding based on regions investigated in previously published TSPO PET studies in people with HIV (Boerwinkle et al. 2020; Coughlin et al. 2014; Garvey et al. 2014; Hammoud et al. 2005; Rubin et al. 2018; Vera et al. 2016; Wiley et al. 2006): whole brain, frontal lobe, occipital lobe, temporal lobe, parietal lobe, amygdala, hippocampus, posterior cingulate gyrus, anterior cingulate, basal ganglia, globus pallidus, striatum, caudate, putamen, thalamus, cerebellum, brainstem, midbrain, pons and medulla. Each ROI was then applied to the dynamic PET data to derive regional time-activity curves.

Model fitting and parameter estimation was performed using the MIAKAT™ pipeline, implemented in MATLAB™ R2019b (The MathWorks, Natick, MA, USA). A two-tissue compartment model using the metabolite-corrected plasma input function was applied to the dynamic PET data using a fixed blood volume correction of 5%. For each ROI evaluated, the main outcome measured was the total volume of distribution (V_T_) estimated from the rate constant as described previously (Gunn et al. 2000). The secondary outcome, distribution volume ratio (DVR), was calculated by normalising the V_T_ at each ROI to cortical grey matter, a pseudo-reference region previously investigated in a [^11^C] PBR28PET study in people with HIV (Vera et al. 2016).

### Statistical analysis

Participant demographics and clinical parameters were described using median (interquartile range) and total (percentage), as appropriate. Differences in variables between participants who started ART during acute HIV infection and chronic HIV infection were analysed using the Mann–Whitney U test and Fisher’s exact test, as appropriate. Difference in variables between participants who started ART during acute HIV infection, participants who started ART during chronic HIV infection and control participants were analysed using the Kruskall-Wallis test. Statistical analyses were conducted using SPSS version 25 (IBM Corp, Armonk, NY, US) and *p*-values < 0.05 were considered statistically significant throughout.

Differences in V_T_ and DVR at the pre-selected ROIs between the participants who started ART during acute HIV infection, participants who started ART during chronic HIV infection and control participants were compared using the Kruskall-Wallis and Mann–Whitney U-tests, as appropriate.

## Results

### Participant characteristics

Participant demographics and clinical parameters are described in Table 1. Seventeen participants living with HIV (9 who initiated ART during acute HIV infection and 8 who initiated ART during chronic HIV infection) and 8 control participants completed [^11^C] PBR28 PET brain imaging and were included. All participants included in the study were male and high-affinity binders on TSPO genotypic testing. Apart from age and bodyweight, no additional demographic or clinical data were available for the control participants.

**Table 1:** Demographics and clinical parameters (where available) of participants who underwent brain [^11^C] PBR28 PET imaging

Variables	Participants who initiated ART during acute HIV infection n = 9	Participants who initiated ART during chronic HIV infection n = 8	**Control participants** n = 8	p-value
Age, years	40 (30, 46)	43.5 (41, 47)	20.5 (20, 25)	0.001^a^
Body weight, kg	74.7 (69.1, 74.9)	82.0 (65.4, 96.4)	79.8 (75.7, 89.2)	0.167^a^
Ethnicity			*not available*	< 0.001
White	6 (66.7)	5 (63)		
Mixed white and Asian	3 (33.3)	0		
Black African	0	2 (25)		
Hispanic	0	1 (12)		
Duration since HIV diagnosis, years	3.3 (2.6, 4.5)	15.0 (3.0, 15.5)	*not applicable*	0.041
Duration on ART, years	3.2 (2.5, 4.4)	4.0 (3.0, 13.5)	*not applicable*	0.052
Duration between HIV diagnosis and ART initiation, weeks	3.6 (3.3, 4.9)	16.0 (7.1, 182.0)	*not applicable*	0.010
Pre-treatment HIV RNA, log_10_ copies/mL	4.4 (3.2, 5.7)	5.3 (4.8, 5.4)	*not applicable*	0.606
Current CD4^+^ T-cell count, cells/µL	687 (652, 1014)	700 (500, 720)	*not available*	0.321
Nadir CD4^+^ T-cell count, cells/µL	586 (370, 687)	228 (160, 250)	*not available*	0.002
Current CD4^+^:CD8^+^ T-cell ratio	1.37 (1.24, 1.42)	0.67 (0.64, 0.82)	*not available*	< 0.001
Current CD8^+^ T-cell count, cells/µL	553 (462, 779)	850 (800, 1380)	*not available*	0.046
ART regimens			*not applicable*	0.011
PI-based	2 (22)	4 (50)		
NNRTI-based	1 (11)	4 (50)		
INSTI-based	6 (67)	0 (0)		

Values are median (interquartile range) or n (%). Differences in variables between participants who initiated ART during acute HIV infection versus chronic HIV infection were analysed using the Mann Whitney U test and Fisher’s exact test, as appropriate, unless stated otherwise

*ART *antiretroviral treatment, *PI *protease inhibitor, *NNRTI *non-nucleoside reverse transcriptase inhibitor, *INSTI *integrase strand transferase inhibitor

^a^Differences in age and bodyweight were analysed between participants who initiated ART during acute HIV infection, participants who initiated ART during chronic HIV infection and control participants using the Kruskall-Wallis test

Overall, the control participants were younger (median (IQR) 21 years (20, 25)) compared with participants who initiated ART during acute HIV infection (median (IQR) 40 years (30, 46)), who were in turn, younger than the participants who initiated ART during chronic HIV infection (median (IQR) 44 years (41, 47)) (Table 1). Of the three groups, participants who initiated ART during acute HIV infection had the lowest median bodyweight (median (IQR) 74.7 kg (69.1, 74.9)).

Participants living with HIV were predominantly of white ethnicity (Table 1). Overall, participants who initiated ART during acute HIV infection versus chronic HIV infection had a shorter duration since HIV diagnosis (median (IQR) 3.3 years (2.6, 4.5) vs 15.0 years (3.0, 15.5), p = 0.04) and shorter duration on ART (median (IQR) 3.2 years (2.5, 4.4) vs 4.0 years (3.0, 13.5), p = 0.05) (Table 1).

Median pre-treatment HIV RNA load was numerically lower in participants who initiated ART during acute HIV infection compared with chronic HIV infection (median (IQR) 4.4 (3.2, 5.7) vs 5.3 (4.8, 5.4) log_10_ HIV RNA copies/mL, p = 0.61) and current CD4^+^ T-cell count was similar between the two HIV-positive groups (Table 1). Participants who initiated ART during acute HIV infection had higher nadir CD4^+^ T-cell count, higher current CD4^+^/CD8^+^ T-cell ratio and lower current CD8^+^ T-cell count compared to participants who initiated ART during chronic HIV infection (Table 1).

At the point when the brain PET-CT scan was performed, the majority of participants who initiated ART during acute HIV infection were on INSTI-based ART regimens (67%), whereas for the participants who initiated ART during chronic HIV infection, equal numbers of participants were on protease inhibitor (PI)-based and non-nucleoside reverse transcriptase inhibitor (NNRTI)-based ART regimens.

### [^11^C] PBR28 total volume of distribution (VT) results

Table 2 describes the mean (standard deviation) [^11^C] PBR V_T_ binding at the selected brain regions, stratified according to the study groups. Overall, no statistically significant differences in [^11^C] PBR28 (V_T_) binding between the three groups of participants at any of the selected regions of interest were observed (unadjusted p-value > 0.05 by the Kruskall-Wallis test for differences between the three groups at all 20 pre-selected ROIs) (Table 2).

**Table 2:** Differences in regional brain [^11^C] PBR V_T_ binding at 20 pre-selected anatomical brain regions of interest between participants who initiated ART during acute HIV infection (aPWH), participants who initiated ART during chronic HIV infection (cPWH) and control participants

**Regions of interest**	**aPWH** **n = 9**	**cPWH** **n = 8**	**CP** **n = 8**	**p-value**
**Whole brain**	3.9 (1.27)	4.4 (1.11)	4.2 (1.16)	0.611
**Frontal lobe**	3.8 (1.29)	4.5 (1.31)	4.3 (1.25)	0.548
**Occipital lobe**	4.0 (1.24)	4.6 (1.06)	4.2 (1.20)	0.398
**Temporal lobe**	4.1 (1.32)	4.4 (1.05)	4.24 (1.14)	0.709
**Parietal lobe**	3.8 (1.20)	4.4 (1.13)	4.4 (1.22)	0.594
**Amygdala**	4.8 (1.71)	5.1 (1.31)	4.9 (1.58)	0.834
**Hippocampus**	4.5 (1.46)	4.9 (1.23)	4.7 (1.34)	0.650
**Posterior cingulate gyrus**	4.1 (1.44)	4.7 (1.39)	4.2 (1.21)	0.552
**Anterior cingulate**	4.1 (1.48)	4.8 (1.54)	4.2 (1.17)	0.552
**Basal ganglia**	3.8 (1.29)	4.4 (1.22)	3.8 (1.10)	0.574
**Globus pallidus**	4.5 (1.83)	5.5 (1.78)	4.4 (1.55)	0.405
**Striatum**	3.7 (1.27)	4.2 (1.14)	3.7 (1.03)	0.532
**Caudate**	3.0 (1.14)	4.3 (2.38)	3.6 (1.15)	0.522
**Putamen**	4.0 (1.31)	4.6 (1.49)	3.9 (1.07)	0.531
**Thalamus**	4.3 (1.73)	5.0 (1.50)	4.8 (1.52)	0.486
**Cerebellum**	4.1 (1.45)	4.6 (1.13)	4.2 (1.14)	0.685
**Brainstem**	4.9 (1.76)	5.3 (1.24)	5.0 (1.32)	0.672
**Midbrain**	5.5 (2.21)	5.7 (1.41)	5.6 (1.35)	0.852
**Pons**	4.8 (1.81)	5.4 (1.30)	4.9 (1.45)	0.572
**Medulla**	5.0 (1.97)	4.7 (0.91)	4.5 (1.26)	0.736

Values reported are mean (standard deviation)

*ROI *region of interest, *ART *antiretroviral treatment, *aPWH *people who initiated ART during acute HIV infection, *cPWH *participants who initiated ART during chronic HIV infection, *CP *control participants, *V*_*T *_total volume of distribution

P-values calculated using the Kruskall-Wallis test

**Fig. 1:**
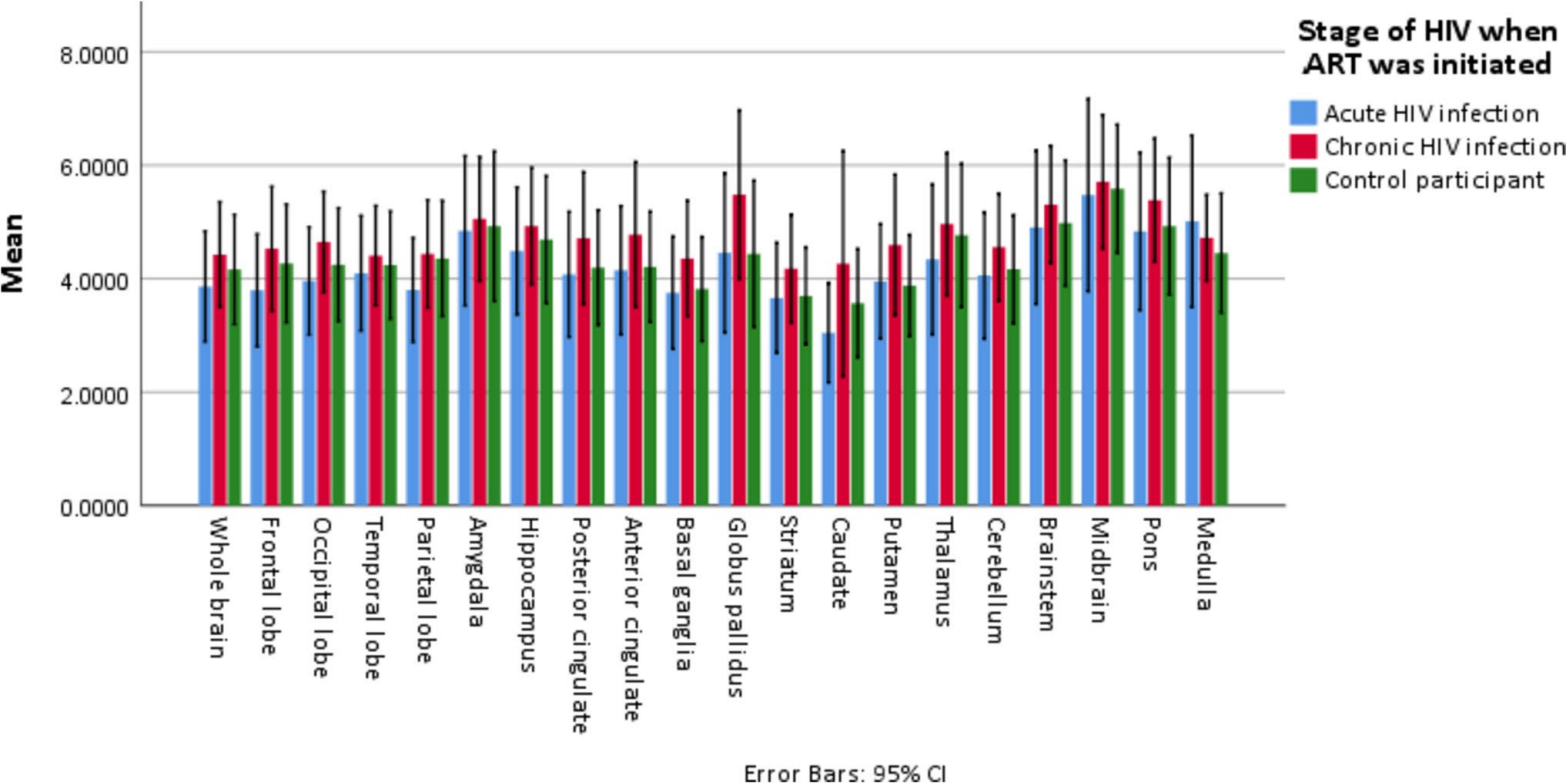
[^11^C] PBR28 V_T_ binding in selected brain regions, stratified according to stage when ART was initiated and control participants

Between the three groups, participants who initiated ART during chronic HIV infection demonstrated a trend towards higher mean [^11^C] PBR28 (V_T_) binding followed by control participants, with participants who initiated ART during acute HIV infection displaying lowest mean [^11^C] PBR28 (V_T_) binding at the majority of the ROIs: whole brain, frontal lobe, occipital lobe, temporal lobe, amygdala, hippocampus, posterior cingulate gyrus, anterior cingulate gyrus, caudate, thalamus, cerebellum, brainstem, midbrain and pons (Table 2 and Fig. 1).

### [^11^C] PBR28 distribution volume ratio (DVR) results

Table 3 describes the differences in [^11^C] PBR28 DVR normalised to cortical grey matter at the 20 pre-selected anatomical brain ROIs between the three groups of participants. We observed no statistically significant differences in [^11^C] PBR28 DVR binding between the three groups of participants at any of the pre-selected regions of interest (unadjusted p-value > 0.05 for differences between the three groups at all 20 ROIs, using the Kruskall-Wallis test) (Table 3).

**Table 3:** Differences in regional brain [^11^C] PBR DVR binding at 20 pre-selected anatomical brain regions of interest between participants who initiated ART during acute HIV infection (aPWH), participants who initiated ART during chronic HIV infection (cPWH) and control participants

**Regions of interest**	**aPWH** **n = 9**	**cPWH** **n = 8**	**CP** **n = 8**	**p-value**
**Whole brain**	0.97 (0.025)	0.97 (0.027)	0.99 (0.022)	0.155
**Frontal lobe**	0.95 (0.058)	0.99 (0.083)	1.00 (0.046)	0.119
**Occipital lobe**	1.00 (0.043)	1.03 (0.066)	1.01 (0.056)	0.759
**Temporal lobe**	1.03 (0.038)	0.97 (0.064)	1.01 (0.017)	0.070
**Parietal lobe**	0.96 (0.055)	0.98 (0.086)	1.04 (0.118)	0.139
**Amygdala**	1.21 (0.076)	1.12 (0.170)	1.17 (0.114)	0.188
**Hippocampus**	1.13 (0.080)	1.10 (0.156)	1.12 (0.061)	0.735
**Posterior cingulate gyrus**	1.02 (0.019)	1.03 (0.090)	1.00 (0.053)	0.505
**Anterior cingulate**	1.03 (0.031)	1.04 (0.085)	1.01 (0.052)	0.215
**Basal ganglia**	0.94 (0.048)	0.96 (0.098)	0.90 (0.031)	0.069
**Globus pallidus**	1.11 (0.277)	1.20 (0.197)	1.02 (0.112)	0.120
**Striatum**	0.91 (0.053)	0.92 (0.093)	0.88 (0.025)	0.103
**Caudate**	0.76 (0.113)	1.00 (0.734)	0.83 (0.138)	0.379
**Putamen**	0.99 (0.055)	1.00 (0.113)	0.93 (0.051)	0.081
**Thalamus**	1.07 (0.080)	1.09 (0.137)	1.11 (0.055)	0.411
**Cerebellum**	1.01 (0.070)	1.01 (0.080)	1.00 (0.037)	0.894
**Brainstem**	1.22 (0.066)	1.18 (0.182)	1.22 (0.065)	0.715
**Midbrain**	1.35 (0.100)	1.27 (0.161)	1.38 (0.090)	0.217
**Pons**	1.20 (0.070)	1.20 (0.202)	1.19 (0.093)	0.676
**Medulla**	1.32 (0.585)	1.06 (0.205)	1.13 (0.117)	0.455

Values are mean (standard deviation)

*ROI *region of interest, *ART *antiretroviral treatment, *aPWH *people who initiated ART during acute HIV infection, *cPWH *participants who initiated ART during chronic HIV infection, *CP *control participants, *DVR *distribution of volume ratio

P-values calculated using the Kruskall-Wallis test

**Fig. 2:**
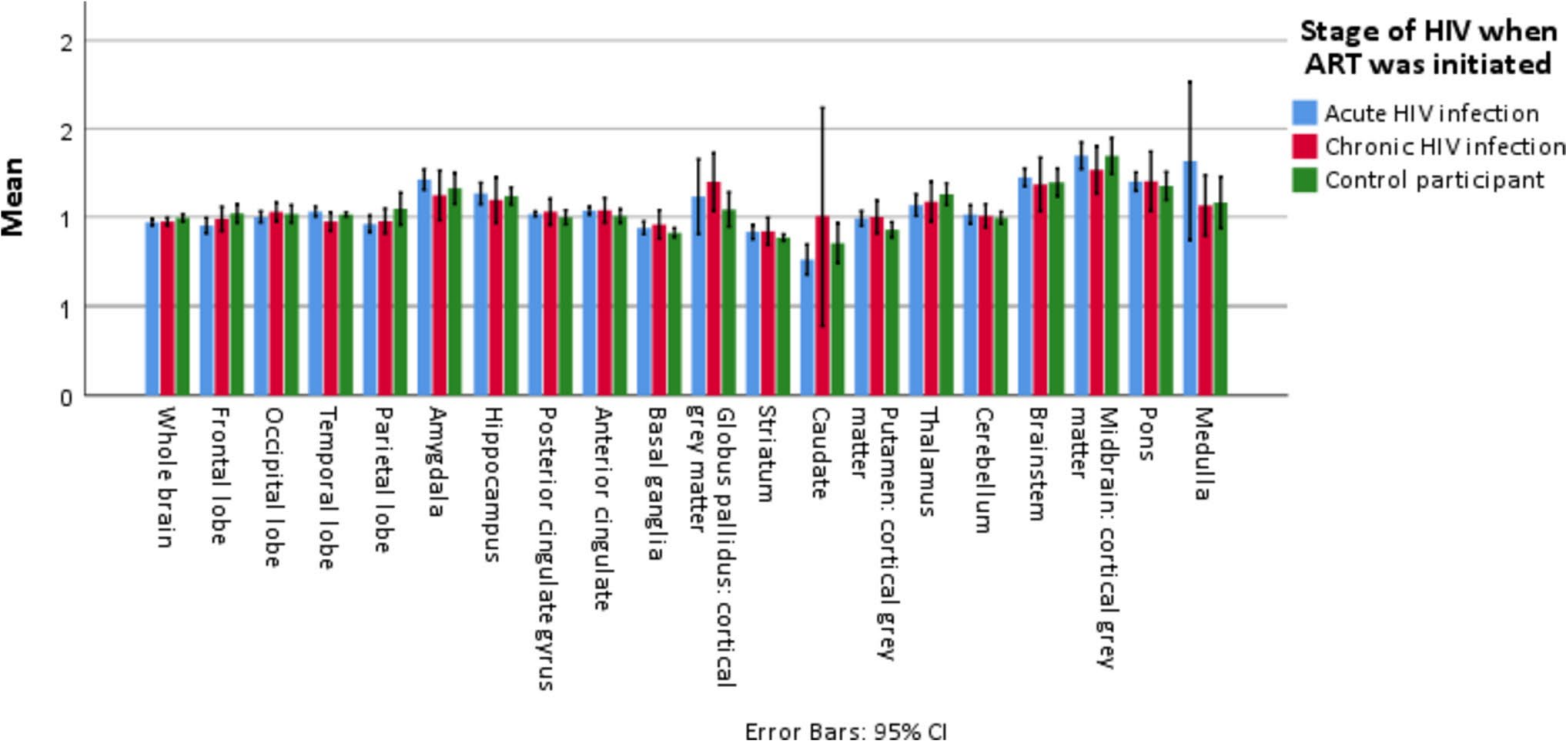
[^11^C] PBR28 DVR binding in selected brain regions, stratified according to stage when ART was initiated and control participants

Mean [^11^C] PBR28 DVR results at the pre-selected ROIs were variable and showed no consistent pattern in trends towards differences between the three groups of participants (Table 3 and Fig. 2).

## Conclusions

This study compares neuroinflammation using [^11^C] PBR28 binding as a proxy in people who initiated ART in acute HIV infection, people who initiated ART in chronic HIV infection and control participants. While no statistically significant differences in [^11^C] PBR28 binding were observed between the three groups, our analyses consistently demonstrated higher absolute mean [^11^C] PBR28 binding amongst people who initiated ART during chronic HIV infection compared with people who initiated ART during acute HIV infection and control participants at the majority of brain anatomical regions studied. Our findings should be interpreted with caution due to the small sample size of our pilot study, however this initial signal may herald a potential true signal that warrants further investigation. The trend towards lower absolute [^11^C] PBR28 binding in participants who initiated ART during acute infection compared with chronic infection corroborates the theory that early ART initiation soon after HIV acquisition may attenuate the neuroinflammatory responses widely reported in persons with HIV who initiated ART during chronic HIV infection (Ulfhammer et al. 2018; Yilmaz et al. 2008).

While some previous TSPO studies in people with HIV have identified statistically significant differences in TSPO binding at certain anatomical locations in people with HIV on ART compared with control participants, we did not observe this in our study. Reasons for the discrepant results between the various TSPO studies in people with HIV include the fact that the previous studies have used different TSPO radiotracers and different methodologies to quantify TSPO binding (Alagaratnam & Winston 2022). Quantification of TSPO binding remains a rapidly evolving field and our study utilised newer models and processes for quantification based on current gold-standard techniques that had not yet been determined at the time when the previous studies were conducted. In a previous study from our group, (Vera et al. 2016) estimated the continuous [^11^C] PBR28 plasma-to-blood ratio using a constant model in the plasma input function. However, we observed that an exponential-approaching-constant model provided a better fit for the data in the majority of the participants in our study. Brain [^11^C] PBR28 data for people who initiated ART during chronic HIV was provided from Vera et al.’s study (Vera et al. 2016), but the same control participants’ data were not available for us to use in our analysis, which may also explain the discrepancy seen between the results of our analyses. Additionally, brain [^11^C] PBR28 signals from grey matter were analysed, as at the time, it was considered the optimal strategy for identifying microglial activation. However, given that HIV disease affects both the grey and white matter throughout the brain, our analyses here included brain [^11^C] PBR28 signals from both grey and white matter. Furthermore, our study is limited by the sensitivity of [^11^C]PBR28; although [^11^C]PBR28 has greater sensitivity than previous TSPO ligands, it may not have sufficient sensitivity to determine differences in ligand binding between the three cohorts of participants in this study.

Whether to use absolute [^11^C] PBR28 binding (V_T_) or [^11^C] PBR28 V_T_ binding normalised to a reference region (DVR) to report regional brain [^11^C] PBR28 binding remains hotly debated. Some studies have suggested a poor test–retest reproducibility with absolute TSPO V_T_ binding due to intra-individual factors and normalisation to a reference or pseudo-reference region can control for unaccounted physiological factors such as stress responses, hormone-mediated changes in TSPO expression, blood cholesterol changes due to food intake and other genotypic factors that may affect TSPO radioligand uptake in the brain (Coughlin et al. 2014; Drugan 1996; Gavish et al. 1999; Jučaite et al. 2012). These factors may impact cerebral radioligand signal in TSPO PET imaging studies and can also cancel out non-binding effects on the TSPO radioligand signals. A study using [^11^C] DPA-713, a second-generation TSPO radioligand, demonstrated improved test–retest reproducibility when [^11^C] DPA-713 VT binding was normalised to a reference region (Coughlin et al. 2014). A major concern with normalising TSPO binding to a reference region is losing the ability to detect changes in the region used as the reference region, by effectively cancelling out the signal in the region of interest by normalising to another region also displaying a signal. Ideally, the reference region chosen should be unaffected by the disease under investigation. However, HIV disease generally affects the whole central nervous system, and a reliable disease-free brain TSPO reference region in people with HIV has not yet been identified. For this reason, we have chosen to use absolute [^11^C] PBR28 V_T_ binding as our primary outcome, with [^11^C] PBR28 DVR binding normalised to cortical grey matter as the secondary outcome. Urgent consensus is required on the optimal methodology for determining [^11^C] PBR28 binding and the optimal reference region for TSPO studies. Until a gold-standard method of measuring TSPO radiotracer uptake is developed and accepted, interpreting and comparing results from TSPO PET neuroimaging studies will remain challenging.

Strengths of our study include the phenotypically well-described cohorts of participants living with HIV. Participants who initiated ART during acute HIV infection were strictly within 3 months of confirmed primary HIV infection. [^11^C] PBR28 binding quantification was performed using the gold-standard methodology using continuous plasma input function via arterial sampling.

On the converse, limitations include the small sample size due to the high cost of performing PET neuroimaging studies, and the control participants who were not demographically matched to the people living with HIV. Overall, participants who initiated ART during chronic HIV infection were older and had higher body weight upon enrolment into the study, which may reflect the normal ageing processes. While control participants were on average younger than both groups of HIV-positive participants, the control participants’ body weight were similar to that seen in participants who initiated ART during chronic HIV infection. To date, published findings on the effect of age on TSPO expression have been inconclusive with some studies demonstrating higher TSPO binding with increasing age and no effect in others (Cagnin et al. 2001; Paul et al. 2019; Rissanen et al. 2018; Schuitemaker et al. 2012; Tuisku et al. 2019). Body mass index has been shown to negatively correlate with brain [^11^C] PBR28 V_T_ binding (Tuisku et al. 2019), however complete body mass index data was not available for our analysis. We are also limited by a lack of data on alcohol use, recreational drug use and smoking history which could be confounders to our findings.

Astrocytes and microglia play very different roles in the central nervous system but emerging data suggests that reactive astrocytes may also have a contributory role to TSPO expression signals (Lavisse et al. 2012) and it is becoming increasingly recognised that [^11^C] PBR28 binding can represent pro-, anti- and mixed inflammatory phenotypes (Owen et al. 2017). Thus, TSPO expression signals in this setting should be interpreted with caution and further studies are warranted to determine the phenotype (pro-, anti- and mixed inflammatory) of the signals identified and the cell type contributing to the [^11^C] PBR28 binding signals (microglia and astrocytes), by correlating brain TSPO binding results with neuropathological specimens with histochemistry and fluid biomarkers.

In summary, this study assessed the effect of early ART initiation soon after HIV acquisition on neuroinflammation using a novel molecular neuroimaging technique. We observed no significant differences in neuroinflammation, using [^11^C]PBR28 binding as a proxy between people who initiated ART during chronic HIV infection, people who initiated ART during primary HIV infection and control participants. A trend towards higher neuroinflammation in people who initiated ART during chronic infection compared with acute infection and control participants was observed, suggesting early ART initiation may reduce neuroinflammation.

## Data Availability

No datasets were generated or analysed during the current study.
